# Therapeutic Potential of Green Synthesized Gold Nanoparticles Using Extract of *Leptadenia hastata* against Invasive Pulmonary Aspergillosis

**DOI:** 10.3390/jof8050442

**Published:** 2022-04-24

**Authors:** Basem M. Abdallah, Enas M. Ali

**Affiliations:** 1 Department of Biological Sciences, College of Science, King Faisal University, Al-Ahsa 31982, Saudi Arabia; eabdelkader@kfu.edu.sa; 2 Department of Botany and Microbiology, Faculty of Science, Cairo University, Giza 12613, Egypt

**Keywords:** gold nanoparticles, *A. fumigatus*, *Leptadenia hastata*, aspergillosis, IPA

## Abstract

Gold nanoparticles are widely used in the biomedical field for the treatment of several diseases, including cancer, inflammatory diseases, and immune system disorders, due to their distinctive physicochemical characteristics. In this study, we investigated the therapeutic potential of green synthesized gold nanoparticles using ethanolic leaf extract of *Leptadenia hastata* (LH-AuNPs) against invasive pulmonary aspergillosis (IPA) in mice. UV/visible spectroscopy, Fourier transform infrared spectroscopy (FTIR), transmission electron microscopy (TEM), X-ray diffraction (XRD), energy-dispersive X-ray spectroscopy (EDX), and zeta potential were used to characterize the biofabricated LH-AuNPs. Antifungal activity of LH-AuNPs was determined by MTT assay, (3-(4,5-dimethyl-2-thiazolyl)-2,5-diphenyl-2H-tetrazolium bromide), time-kill assay, and radial growth inhibition. TEM and SEM were used to examine the mode of the antifungal action of LH-AuNPs. The in vivo activity of LH-AuNPs against IPA was studied using a well-established IPA mouse model. LH-AuNPs excreted antifungal activity against *Aspergillus fumigatus* with MIC 64 µg/mL and inhibited the radial growth of *A. fumigatus* by 30% compared to the control. LH-AuNPs caused distortion and collapse of fungal hyphae and deterioration of cell walls. Interestingly, LH-AuNPs did not display any cytotoxicity on cultured primary bone marrow stem cells (BMSCs) or A549 human lung cell line in vitro at MIC concentration. IPA mice treated with LH-AuNPs displayed significant lung tissue repair without any in vivo cytotoxicity. LH-AuNPs administration showed significant suppression of fungal burden and gliotoxin production in the lung. In addition, LH-AuNPs inhibited IPA-induced pro-inflammatory cytokines production, including interleukin-1 (IL-1), interleukin-17 (IL-17), and tumor necrosis factor-alpha (TNF-α), and reduced oxidative stress in lung. In conclusion, our data provide LH-AuNPs as a novel nanoparticle therapy for IPA.

## 1. Introduction

The incidence of life-threatening mycotic infections caused by *Aspergillus* species and other fungi has increased in the past few years [1,2]. *A. fumigatus* is one of the most common fungal pathogens that can cause a variety of diseases, such as invasive pulmonary aspergillosis (IPA), aspergilloma, and allergic syndromes [3]. The clinical spectrum of patients who are at high risk for IPA is extensive. Patients getting allogeneic bone marrow transplants and patients with hematological malignancy have a higher risk of IPA [4]. 

Owing to the complications in treating IPA, it remains challenging to increase novel insights into the distribution of infection. The antifungal drugs used for the treatment of IPA are limited due to the eukaryotic nature of fungal cells; they have only a limited set of specific targets that do not overlay with their mammalian counterpart. The currently available antifungal agents have several associated problems. Amphotericin B (AMB) can cause severe adverse effects because of its nephrotoxicity [5]. Although lipid preparations of AMB have decreased nephrotoxicity, renal injury is still detected, and infusion-related toxicity might be debilitating. Itraconazole is not absorbed in adequately higher amounts to be therapeutic and might interact harmfully with an extensive spectrum of drugs [6].

Therefore, there is a critical necessity for the development of novel antifungal drugs with novel modes of action [7]. Gold nanoparticles have been the focus of increasing interest in many biomedical applications due to their mechanical, chemical, thermal, optical, and biocompatibility properties. These applications include the use of AuNPs in diagnostic tracers, biosensing, photothermal and radiotherapy, and cell imaging [8,9,10]. In addition, the approval of using AuNPs by the Food and Drug Administration (FDA) increased the applications of AuNPs as therapeutic agents in nanomedicine, such as cancer therapy and drug carriers [11,12].

Green synthesis of nanoparticles using biological extracts has been developed to provide large-scale production, where mild reaction conditions without additional reducing agents and stabilizers were applied that minimize the hazardous wastes and reduce the costs of nanoparticle production [13]. We have recently reported that the green synthesis of silver nanoparticles (AgNPs) using plant extracts could effectively produce nanoparticles with antimicrobial effects against *C. albicans* and *A. fumigatus* in vitro and in vivo [14,15]. 

*L. hastata* is a member of the family *Asclepiadaceae*, used as a vegetable, and is considered a scarce food due to its higher content of beneficial nutrients [16,17]. It is used in herbal medicine to treat diabetes [18] and has anti-inflammatory and antitrypanosomal activities [19]. It is also applied as an antimicrobial agent against several fungal and bacterial species [20,21]. In this study, we explore the antifungal action of biosynthesized AuNPs using leaf extract of *L. hastata* against *A. fumigatus* in vitro and in vivo. LH-AuNPs showed antifungal activity against *A. fumigatus* in vitro and therapeutic effect for IPA in vivo via reducing fungal burden, gliotoxin production, and IPA-induced pro-inflammatory cytokines.

## 2. Materials and Methods

### 2.1. Preparation of Plant Extract

*L. hastata* was identified taxonomically and authenticated by the Department of Botany and Microbiology, Cairo University, where a voucher specimen was deposited (Voucher number N8F5). Then, 10 g of dried *L. hastata* leaves powder was suspended in 50 mL of 95% ethanol for 24 h at 37 °C. The extract was filtered and evaporated by a rotary vacuum evaporator at 40 °C and stored at 4 °C.

### 2.2. Green Synthesis of LH-AuNPs 

Five mL of the plant extract was added to 45 mL of 1 mM aqueous HAuCl4 solution. The change of color from pale yellow to vivid ruby-red demonstrates the reduction of AuCl_4_ and the formation of AuNPs. The suspension was centrifuged at 3500 rpm for 10 min to remove the unreacted plant extract. The biosynthesized nanoparticles were collected by centrifugation at 12,000 rpm for 20 min and purified by washing with sterile distilled water to obtain nanoparticles in pellet form. The purified AuNPs were then suspended in distilled water for further study. 

### 2.3. Characterization of LH-AuNPs

The reduction of gold ions was confirmed by measuring the UV-vis spectrum of the reaction mixture. The spectral analysis was done using a UV-1602 Double Beam UV/Vis spectrophotometer at a resolution of 1 nm from 300 nm to 800 nm. The selected ratio was allowed to freeze-dry for further characterizations. The crystalline pattern of powdered AuNPs was recorded by XDL 3000 powder X-ray diffractometer. The Fourier transform infrared (FTIR) spectra were performed on a Perkin Elmer spectrum instrument at a resolution of 4 cm^−1^ in KBr pellet. The morphology, purity, structure, and elemental distribution of the LH-AuNPs were observed by transmission electron microscopy (TEM) and energy-dispersive X-ray spectroscopy (EDX). TEM images were obtained by placing droplets of the purified NPs suspended in water on a carbon-coated copper grid and drying them at room temperature before microscopic analysis. EDX was done by Energy Dispersive X-ray analysis “EDX”. A double-sided carbon coated glass cover slip was used, the lower side of which was used to be fixed to the stab, while, on upper side, sample was loaded and was examined by EDX (Oxford, UK, Model No. INCA 200). The zeta-potential measurements were performed with a Zetasizer Nano ZS (Malvern Instruments) in a disposable cell at 25 °C, using Zetasizer 7.01 software. Zeta potential is used to study AuNPs stability. These measurements were carried out first 2–3 h after the LH-AuNPs were synthesized and then once a week for 3 weeks. Between measurements, all LH-AuNPs suspensions were kept frozen at −18 °C. The suspension was allowed to melt and equilibrate at room temperature for 2 h prior to measurement. Straight away after the measurements were taken, the extracts were frozen again.

### 2.4. Microorganism and Culture Conditions

A strain of *A. fumigatus* was formerly isolated by our group from an immunocompromised patient with IPA [14]. Fungal suspension was prepared by cultivating *A. fumigatus* on Peptone Yeast Extract Agar (PYG) medium for 5 days at 35 °C, and the conidia were harvested as described previously [22]. 

### 2.5. Antifungal Susceptibility Test

Antifungal assay was carried out by 3-(4,5-dimethyl-2-thiazolyl)-2,5-diphenyl-2H-tetrazolium bromide (MTT) method with slight modifications [23]. The conidia were counted by using a hemocytometer, adjusted to a density of 10^6^ CFU/mL; 96-well flat-bottom plates were used, and 90 μL of RPMI 1640 and 100 μL from LH-AuNPs were added into the first wells. Then, LH-AuNPs were serially diluted to final concentrations ranging from 2 to 256 μg/mL. In each well of the column, aliquots of 10 μL of the *A. fumigatus* inoculum were dispensed. A positive control was prepared in the same way with the standard antifungal drug amphotericin B (AMB). After 24 h of incubation, 20 µL of an MTT was added to each well. The plates were then incubated at 37 °C for 48 h. After incubation, the formazan assay product was extracted, and the optical density at 540 nm was measured. Minimum inhibitory concentration (MIC) was defined as the lowest concentration of the drug that resulted in 100% visual inhibition of growth. For the determination of radial growth, the spores of *A. fumigatus* were added to the center of potato dextrose agar (PDA) plates containing sub-MIC of LH-AuNPs (32 µg/mL). DMSO was used as negative control. The plates were incubated at 35 °C, and growth of *A. fumigatus* was observed after 7 days. The micrographs were obtained using an Olympus Inverted Microscope IX50 equipped with a Lumenera Infinity camera (Olympus Corporation, Tokyo, Japan).

### 2.6. Pigment Inhibition

Conidial suspensions of *A. fumigatus* (1 × 10^5^) were inoculated in PDA plates containing sub-MIC of LH-AuNPs (32 µg/mL) and incubated at 35 °C. After 72 h, the morphological examination of the colonies was performed.

### 2.7. Time–Kill Curve Studies 

Briefly, 10 mL of *A. fumigatus* conidial suspension (1 × 10^5^ conidia/mL) was added to 10 mL of RPMI-1640 medium (negative control) with AMB (positive control) or LH-AuNPs. Cultures were agitated at 37 °C. At different time intervals (0, 4, 8, 12, 16, and 24 h), a sample of 0.1 mL was removed from each test suspension, spread on PDA plates, and then incubated at 37 °C for 48 h. The time–kill curves were made by plotting the colony-forming units (CFU) per milliliter surviving at each time interval in the presence of various antimicrobial drugs.

### 2.8. Morphological Modifications

#### 2.8.1. Scanning Electron Microscopy (SEM) 

The LH-AuNPs-treated *A. fumigatus* mycelia sections were collected, fixed with formaldehyde, washed with phosphate buffer solution, dehydrated with an alcohol solution, and then submitted to critical point drying as described [24]. The fungal mycelia were prepared for SEM using JEOL (JSM6380 LA) instrument.

#### 2.8.2. Transmission Electron Microscopy (TEM)

*A. fumigatus* mycelia treated with LH-AuNPs were observed using TEM. Saline-treated mycelia were used as a control. All samples were infiltrated with 1:1 then 1:2 ratios of ethanol to resin in a vacuum overnight. The samples were then fixed and allowed to evacuate overnight, then placed in an oven to polymerize for four days. The samples were trimmed and thin sectioned. The sections were post-stained with 7.5% uranyl acetate and Reynolds’s lead citrate. TEM images of the samples were taken using the JEM-1210 TEM instrument (JEOL USA Inc., Peabody, MA, USA) and operated at 90 kV [25].

### 2.9. In Vivo Experiment Design

The procedure of in vivo IPA experiment was approved by the Cairo University, Faculty of Science Institutional Animal Care and Use Committee (IACUC) (Egypt), (CUFS/F/10/13). Swiss albino mice (10 weeks) were obtained from the animal house, National Research Center. Mice were housed at a controlled temperature of 25 ± 2 °C and 12 h dark/light cycle with a standard diet and water ad libitum. Thirty male mice were grouped into 3 groups (*n* = 10), and neutropenia was induced by a single intraperitoneal (ip) administration of cyclophosphamide (150 mg/kg) as described previously [26]. Three days post neutropenic induction, 20 of the neutropenic mice were infected with conidia of *A. fumigatus* (1 × 10^5^) (administered by intranasal instillation), as described [27]. Mice groups were assigned as follows: group 1 (control): control mice injected intravenously with Hank’s Balanced Salt Solution (HBSS); group 2 (IPA control): IPA mice intravenously injected with HBSS after 24 h of fungal inoculation; group 3 (LH-AuNPs group): IPA mice intravenously injected with one injection of LH-AuNPs (0.5 μg/g) after 24 h of fungal inoculation [28]. 

### 2.10. Measurement of Fungal Burden in Lung Tissue

Lungs were dissected from IPA mice after euthanization, weighed, and homogenized in 2 mL of sterile saline with sterile tissue grinders. Numbers of CFU were determined by performing serial dilutions of the homogenized tissue in sterile saline and plating 50 g of lung homogenate on 110 mm diameter potato dextrose agar plates. Plates were incubated at 37 °C, and numbers of CFU were counted at 24 and 48 h.

### 2.11. Measurement of Gliotoxin 

Lung tissues were soddened in plastic bags and mixed with 5 mL of water. The samples were then homogenized, and 10 mL HCl was added to homogenates on a shaker for 30 min. Supernatants of cell cultures were exposed to solid-phase extraction by loading 1 mL of each sample solution onto an Oasis HLB cartridge (Waters Corporation, Milford, MA, USA), which was conditioned with 2 mL of methanol–water mixture. The cartridge was then washed with 1 mL of 5% methanol in water, and the analyte was eluted with 1 mL of absolute methanol. For HPLC analysis, the extract was evaporated to dryness at 50 °C; the residue was added to 0.2 mL methanol and used for the gliotoxin measurement according to the method of [29]. Briefly, 20 µL of the sample was injected at a flow rate of 1 mL min^−1^. Gliotoxin was eluted from the HPLC column after 20 min, and the concentrations were measured by interpolation from a calibration curve (25–1000 ng mL^−1^) prepared using the gliotoxin powder.

### 2.12. Cell Culture and Cytotoxicity Assay

The A549 human lung carcinoma epithelial-like cell line was obtained from ATCC (#CCL-185). Cells were cultured in DMEM supplemented with 10% Fetal Bovine Serum Heat Inactivated (FBS) (Gibco Invitrogen, Waltham, MA, USA) and 1% penicillin/streptomycin (P/S) (Gibco Invitrogen, USA). Primary mouse bone marrow-derived mesenchymal stem cells (BMSCs) were isolated from 2-month-old C57BL/6J mice, as described previously [30]. Cells from bone marrow were suspended in PBS and filtered through a 70 μm filter. Isolated cells were cultured in RPMI-1640 medium supplemented with 1% penicillin/streptomycin (P/S) and 12% FBS (Gibco Invitrogen, USA). Non-adherent cells were collected after 24 h by centrifugation and re-cultured in a fresh medium. 

For cytotoxicity assay, cells were treated with different concentrations of LH-AuNPs in 96-well plates for 48 h, and an MTT cell proliferation assay kit (Sigma-Aldrich, Darmstadt, Germany) was used to measure the cell viability. Cells were incubated with a medium containing 0.5 mg/mL MTT to metabolize to formazan. Optical density was measured at 550 nm using an ELISA plate reader [31]. Values were expressed as a percentage of control non-treated cells.

### 2.13. Histological Study

The lung was fixed in 10% buffered formalin, embedded in paraffin, sectioned, and stained either with hematoxylin and eosin (H & E) or periodic acid–Schiff stained (PAS). Nikon 80i light microscope (Nikon Corporation, Tokyo, Japan) was used to take tissue sections imaged using.

### 2.14. Biochemical Assays

For kidney and liver function assays: serum biochemical markers including aspartate aminotransferase (AST) activity, alanine aminotransferase (ALT), urea, and creatinine were measured according to the instruction manual of commercially available kits from (Abcam, Cambridge, UK).

For antioxidant enzyme measurements: lung tissues were homogenized in HBSS, incubated in ice bath, and centrifuged at 12,000 r/min for 15 min at 4 °C. The supernatants were collected for measurements. Catalase (CAT) activity was determined by measuring the decrease in absorbance of hydrogen peroxide at 240 nm following the method of [32]. Superoxide dismutase (SOD) activity was determined using the adrenochrome test, which relies on the ability of SOD to inhibit the autoxidation of epinephrine in alkaline according to the method [33], and malondialdehyde (MDA) level was measured by the thiobarbituric acid test [34]. 

For pro-inflammatory cytokines assays: TNF-α, IL-1, and IL-17 were measured by ELISA kit Assay (MyBioSource, Inc., San Diego, CA, USA) according to the manual instructions. 

### 2.15. Statistical Analysis

All values are expressed as mean ± SD (standard deviation) of at least 3 independent experiments. Power calculation was performed for 2 samples using an unpaired Student’s *t*-test (2-tailed), assuming equal variation in the two groups. Differences were considered statistically significant at * *p* < 0.05 and ** *p* < 0.005.

## 3. Results

### 3.1. Biosynthesis and Characterization of LH-AuNPs

**Figure 1:**
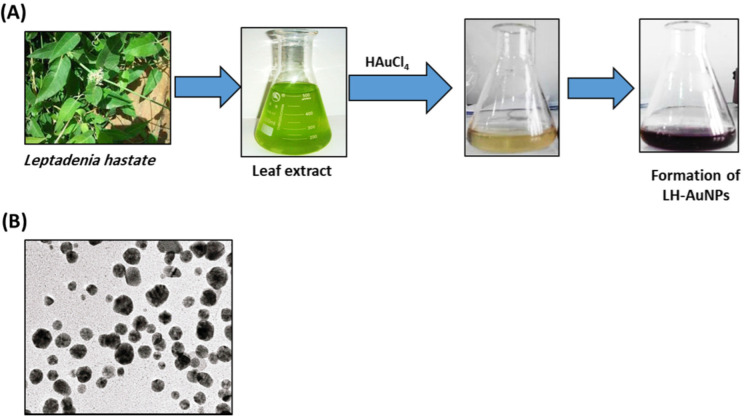
Biosynthesis of LH-AuNPs using *L. hastate* leaf extract. (**A**) The biosynthesis of LH-AuNPs was performed by combining *L. hastate* leaves powder (10 g) with 50 mL of 95% ethanol for 24 h at 37 °C in a 200 mL Erlenmeyer flask; 5 mL of the plant extract was added to 1 mM aqueous HAuCl4 solution (45 mL). The solution changed color from pale yellow to vivid ruby-red, signifying the formation of AuNPs. (**B**) TEM image of LH-AuNPs showed spherical or hexagonal shapes with lattice fringes s with sizes ranging from 5 to 30 nm.

**Figure 2:**
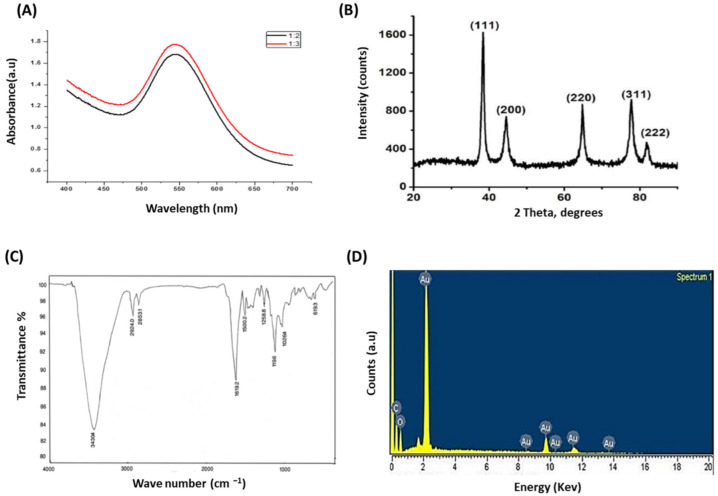
Confirmation of biosynthesized LH-AuNPs. (**A**) UV–Vis spectrum of LH-AuNPs. Different ratios give different absorption peaks, but the most extreme peak with maximum absorbance was recorded 1:2 on 544 nm. (**B**) XRD spectrum recorded for LH-AuNPs showed clear peaks of cubic phases at 38.2 (111), 44.3 (200), 64.9 (220), 77.5 (311), and 81.5 (222), which confirms the crystalline nature of AuNPs. (**C**) FTIR spectrum of LH-AuNPs exhibited two peaks related to OH/NH and C=O groups. The presence of OH group could be ascribed to peak at 3444.6 cm^−1^. The peak of 1732.0 corresponded to C=O group. (**D**) EDX spectrum of LH-AuNPs shows strong signals in the gold region and confirms the formation of gold nanoparticles. A strong peak was displayed around 2.40 keV, which is the characteristic of gold nanoparticles.

LH-AuNPs were biosynthesized from an ethanolic leaf extract of *L. hastate.* The color of *L. hastate* extract after the addition of aqueous chloroauric acid changed from pale yellow to vivid ruby-red (Figure 1A). This red color indicated the formation of AuNPs. The biosynthesis of LH-AuNPs was confirmed by UV/Vis spectrum. Different concentrations (1:2 and 1:3) were used for the optimization of AuNPs. Both ratios showed surface plasmon resonance (SPR) peaks in the range of 500–600 nm, which is specific for AuNPs. The most extreme peak with maximum absorbance was recorded at 1:2, which showed an SPR peak at 544 nm (Figure 2A). TEM revealed a majority of spherical or hexagonal shapes with lattice fringes nanoparticles and sizes ranging from 5 to 30 nm (Figure 1B). The XRD results showed clear peaks of cubic phases at 38.2 (111), 44.3 (200), 64.9 (220), 77.5 (311), and 81.5 (222), which confirms the crystalline nature of AuNPs (Figure 2B). 

FTIR spectra of *L. hastate* extract were expressed in Supplementary Figure S1. Taking the spectrum of plant extract as control, the involvement of different functional groups of *L. hastate* extract in the reducing and stabilizing process of nanoparticles synthesis was evaluated. Absorbance bands at 3430.5 could be due to the O-H stretching vibration of the phenol groups, which might be responsible for the formation and stabilization of nanoparticles. The peak of 1627.9 cm^−1^ corresponded to the C=O group. However, the major absorption peak in the FTIR spectra of synthesized AuNPs is related to OH/NH and C=O groups. The presence of OH group could be ascribed to a peak at 3444.6 cm^−1^. The peak of 1732.0 cm^−1^ corresponded to the C=O group (Figure 2C). The main absorbance band of LH-AuNPs slightly shifted in comparison with the control spectrum. This shifting revealed that biomolecules present in plant extract were responsible for the reduction in gold salt. EDX spectrum of the biosynthesized AuNPs showed strong signals in the gold region that confirmed the formation of AuNPs. A clear, strong peak was observed around 2.40 keV, which is a characteristic of gold nanoparticles (Figure 2D). There were also some weak signals for carbon and oxygen atoms, which might be due to the X-ray emittance from the enzymes/proteins of the biomolecules involved in the formation and capping of gold nanoparticles. Zeta-potential values are often used as a mark indicative of the stability of colloidal particles. The absolute values replicate the net electrical charge on the particles’ external surface that arises from the surface functional groups. Nanoparticles are considered to exist as stable colloids if their zeta potential is more than 25 mV or less than −25 mV [35]. The zeta potential of the LH-AuNPs was −26.1 mV ± 0.2 mV; the suspension of LH-AuNPs in a buffer formed a stable colloid (well-dispersed) with no visible aggregation over 6 months (Supplementary Figure S2).

### 3.2. In Vitro Antifungal Activity of LH-AuNPs against A. fumigatus

**Figure 3:**
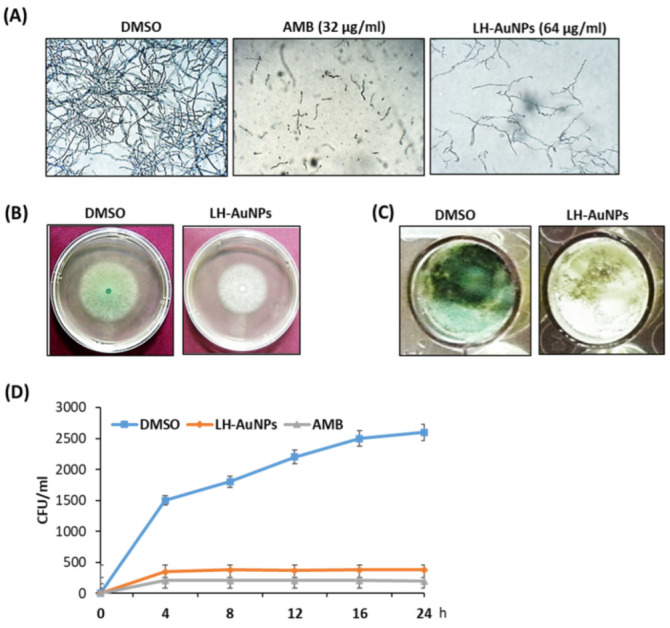
Antimicrobial potential of LH-AuNPs. (**A**) Inverted microscope images of *A. fumigatus* treated with DMSO, LH-AuNPs, and AMB at their MIC values. Visual alterations in mycelial growth are obvious at three different treatments. (**B**) Radial growth of *A. fumigatus* was inhibited, where LH-AuNPs reduced the relative radial growth of *A. fumigatus* by 30% compared to the control. (**C**) Pigment formation defects after LH-AuNPs treatment. The LH-AuNPs treated colonies lacked green pigmentation, signifying they formed few conidia. (**D**) Time–kill curves of *A. fumigatus* following exposure to LH-AuNPs and AMB. Values are mean ± SD of three independent experiments.

We examined the antifungal activity of AMB and LH-AuNPs against *A. fumigatus* using an MTT assay. After 48 h of incubation, the results showed that, with increasing concentrations, the growth of *A. fumigatus* was significantly inhibited. At concentrations greater than 32 and 64 μg/mL, no fungal colonies were visible in the case of AMB and LH-AuNPs, respectively (Figure 3A). Thus, we concluded that the MIC of LH-AuNPs was 64 μg/mL. The inhibitory effect of AMB and LH-AuNPs was also observed using inverted microscopy. A strong visual difference in mycelia density and growth was detected among treatments (Figure 3B). Treatment with 64 μg/mL of LH-AuNPs severely decreased mycelial growth when compared to control. Radial growth of *A. fumigatus* was repressed by treatment with LH-AuNPs, and 64 μg/mL of LH-AuNPs decreased the relative radial growth by 30% as compared to the control (DMSO). The treated colonies appear to be deficient in green pigmentation, suggesting the formation of little or no conidia (Figure 3B). Additionally, these phenomena were also detected in liquid culture, where the green pigmentation was completely deficient after incubation with LH-AuNPs (Figure 3C). The time–kill curves displayed the fungistatic action of both AMB and LH-AuNPs at 32 and 64 µg/mL, respectively, on the growth of *A. fumigatus* cells (Figure 3D). After only 8 h of incubation, LH-AuNPs completely inhibited the growth of *A. fumigatus* to zero colonies (Figure 3D).

### 3.3. Ultrastructural Analysis of the Interaction between LH-AuNPs and A. fumigatus Cells Using TEM and SEM

**Figure 4:**
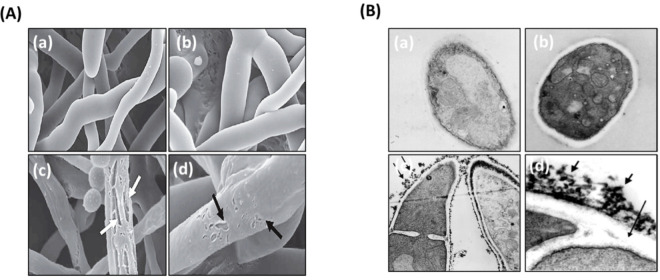
Electron microscopy photographs of *A. fumigatus* after treatment with LH-AuNPs. (**A**) SEM images of *A. fumigatus* treated with saline (**a**,**b**) and LH-AuNPs (**c**,**d**) (64 μg/mL). Black arrows specify pitting and tearing destruction to the cell wall. White arrows show penetration of cell wall into the cytoplasm. Bar = 5 μm. (**B**) TEM images of *A. fumigatus* treated with saline (**a**,**b**) showing normal growth of *A. fumigatus* hyphae and treated with LH-AuNPs (**c**,**d**) showing reticular accumulations on the cell wall on the outer fibrillar layer (arrows) (**c**) and the outer fibrillar layer has a lattice-like structure that is thready (thick arrows). The inner fibrillar layer is not consistently observable (thin arrow) (**d**).

The effect of LH-AuNPs (64 μg/mL) on *A. fumigatus* hypha was studied using SEM. Untreated filaments were smooth and intact with an identical width (Figure 4A(a,b)). Conversely, variable degrees of cell wall deterioration and damage were observed after treatment with LH-AuNPs. The fungal cell wall exhibited severe pitting, tearing, and penetration in the cytoplasm, with the indication of irregular and rough cell walls, and widespread blebbing. In addition, the hyphae looked distorted, shrunken, and lost structure and rigidity (Figure 4A(c,d)).

The TEM revealed that untreated hypha of *A. fumigatus* displayed cells with normal and distinct organelles (Figure 4B(a,b)). The fungal cell membrane seemed sharp and electron-dense. The outer fibrillary layer showed a lightly distributed, rough, electron-dense layer on the cell wall surface. Conversely, there were prominent alterations in the cell wall, cell membrane, and cytoplasm in LH-AuNPs treated cells (Figure 4B(c,d)). The inner granular layer of the cell wall was severely disrupted. Aggregates of the outer surface of the cell wall appeared, and a marked disruption of the integrity of the outer cell wall was also observed.

### 3.4. LH-AuNPs Show No In Vitro Cell Toxicity on Animal and Human Cells 

**Figure 5:**
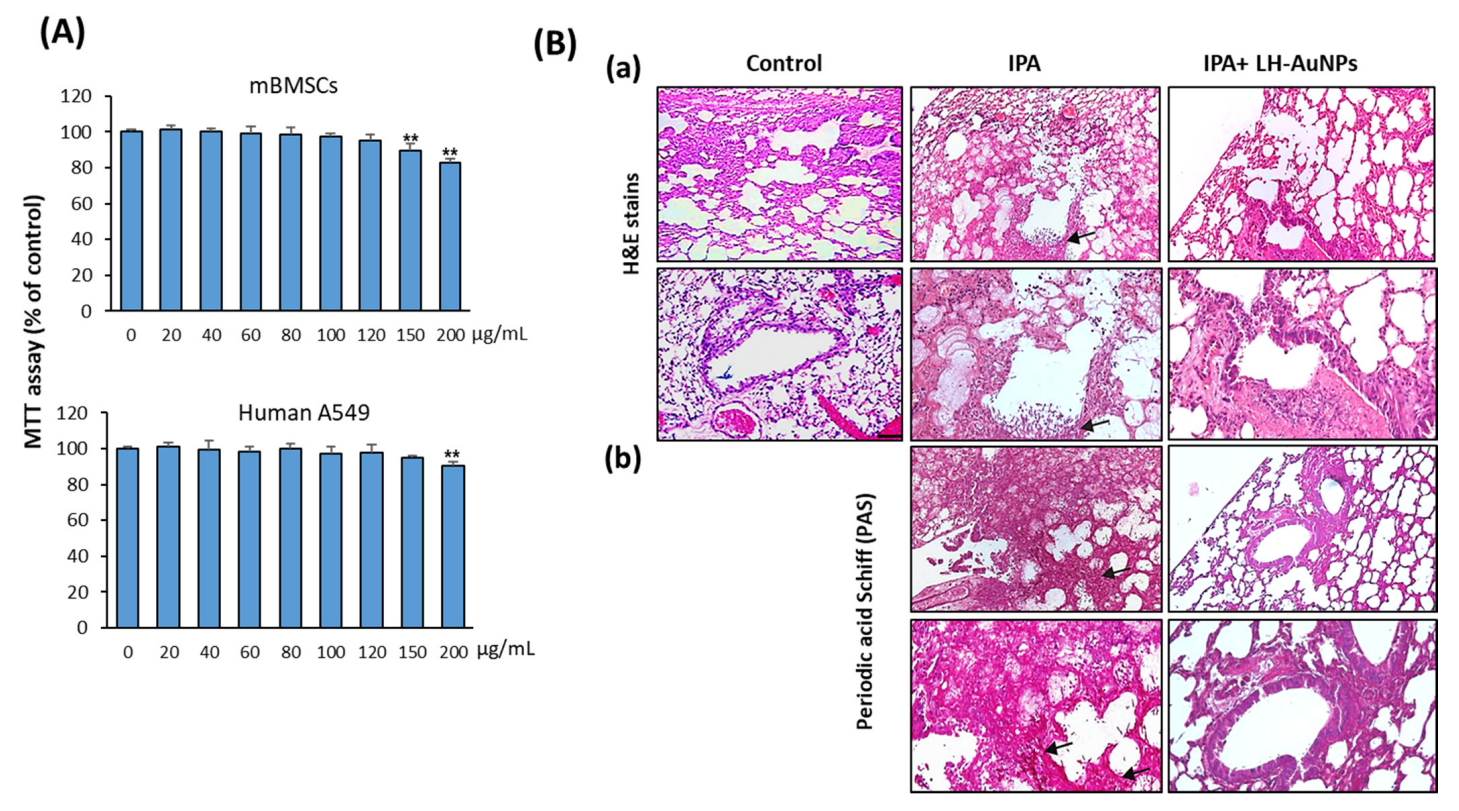
LH-AuNPs repair lung tissue damage in IPA mice. (**A**) Cytotoxicity of LH-AuNPs on human lung cancer cell line, A549, and primary mBMSCs. The dose-dependent effect of LH-AuNPs on cell viability was measured by MTT assay after 48h of treatment. Values are mean ± SD of three independent experiments (** *p* < 0.005, compared to control non-treated cells). (**B**) Histological analysis of lung tissues (3 days post-LH-AuNPs treatment) from control, IPA-non-treated, and IPA-treated mice with LH-AuNPs. Sections stained with H & E (**a**) and periodic acid–Schiff (PAS) (**b**). Extensive fungal growth and tissue damage are evident in the non-treated IPA mice. Arrows indicate fungal balls with great density fungi and proliferating hyphae, while there is a lack of fungal balls and hyphae in the lungs of animals with LH-AuNPs treatment.

We further examined the cytotoxicity of LH-AuNPs on human lung cancer cell line, A549, and primary mBMSCs using cell viability MTT assay. LH-AuNPs showed no cytotoxicity on mBMSCs and human A549 cells up to the concentration of 120 and 150 μg/mL, respectively. A significant reduction in cell viability started to be observed at concentrations of 150 and 200 µg/mL on mBMSCs and human A549 cells, respectively (Figure 5A). Thus, LH-AuNPs showed very low cytotoxicity on animal cells up to the two folds of the MIC concentration (64 μg/mL).

### 3.5. LH-AuNPs Effectively Repair Lung Tissue Damage in IPA Mice without Any In Vivo Toxicity 

As shown in Figure 5B, compared to control mice, the IPA mice displayed severe pulmonary lesions described by multifocal infiltrations of macrophages and neutrophils connected with vascular phenomena (necrosis and hemorrhages) and necrosis of alveolar and bronchiolar epithelial cells (Figure 5B(a)). In addition, a higher number of proliferating hyphae and coagulation necrosis were also detected in the blood vessels, bronchioles, and alveoli, as revealed by the stained section with PAS (Figure 5B(b)). Interestingly, treatment of IPA mice with LH-AuNPs showed to significantly repair the lung tissue damage, as revealed by less extensive inflammation in H & E staining of lung tissues of LH-AuNPs-treated mice (Figure 5B(a)). Moreover, PAS staining confirmed the lack of fungal infection when LH-AuNPs were used (Figure 5B(a)). 

**Figure 6:**
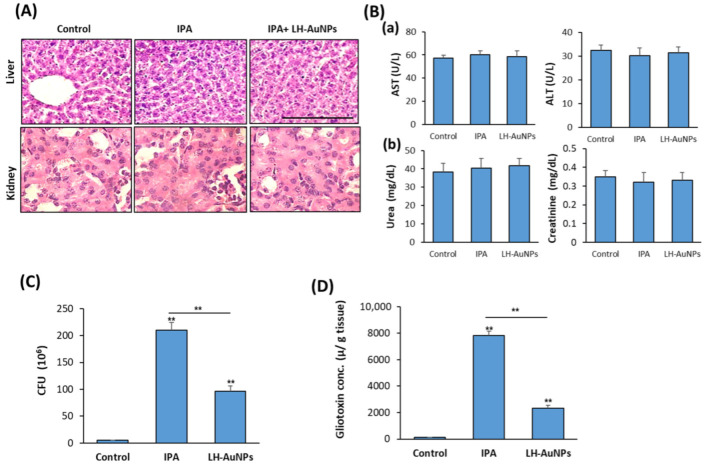
LH-AuNPs suppress fungal burden and gliotoxin production in lung of IPA mice. In vivo cytotoxicity of LH-AuNPs in IPA mice. (**A**) H & E histological sections of liver and kidney from control, IPA-non-treated, and IPA-treated mice with LH-AuNPs. (**B**) Serum biochemical markers of liver function (**a**) AST and ALT and (**b**) renal function, including urea and creatinine. Biochemical analysis was performed after 3 days of treatment with LH-AuNPs. (**C**) Effect of LH-AuNPs on fungal load in lung homogenate of IPA mice. (**D**) Measurements of lung gliotoxin concentration in IPA mice after 3 days of LH-AuNPs. Values are expressed as means ± SD (n = 10 mice/group) (** *p* < 0.005, compared to control non-treated mice).

In association with the therapeutic effect of LH-AuNPs in IPA mice, LH-AuNPs did not show any toxicity or damage to the liver or kidney, as mentioned by H & E histological analysis (Figure 6A). Moreover, biochemical analysis of serum markers for liver function (AST and ALT) and kidney function (Urea and creatinine) were normal and not affected by LH-AuNPs treatment (Figure 6B).

### 3.6. LH-AuNPs Excert Significant Reduction of A. fumigatus Colonization and Gliotoxin Production

To measure the efficiency of LH-AuNPs against *A.* *fumigatus* infection in IPA mice, we determine the mean fungal burden in the lung of IPA-treated mice against control, IPA-non-treated mice. As shown in Figure 6C, the mean burden of fungal cells in the lung was 96 × 10^6^ in LH-AuNPs versus IPA 210 × 10^6^ in IPA-non-treated mice (Figure 6C). In addition, LH-AuNPs significantly reduced the gliotoxin production by *A. fumigatus* in IPA mice by 69% as compared to the IPA mice (Figure 6D). 

### 3.7. LH-AuNPs Significantly Reduce Inflammation and Oxidative Stress in IPA Mice

**Figure 7:**
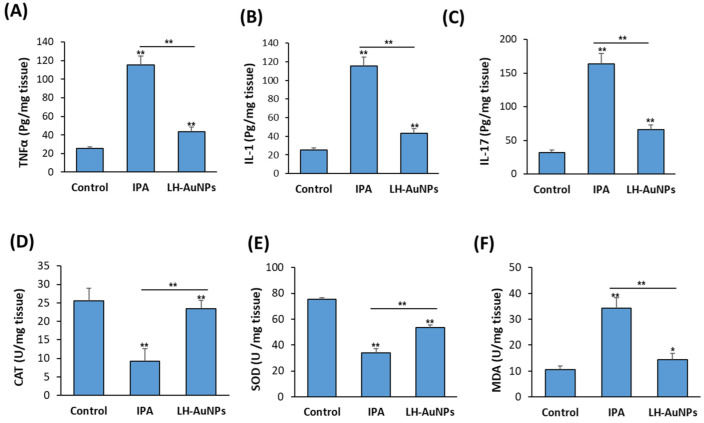
Inhibitory effect of LH-AuNPs on pro-inflammatory cytokines production and oxidative stress in IPA mice. Measurements of inflammatory cytokines, (**A**) TNF-α, (**B**) IL-1, and (**C**) IL-17 after 3 days of LH-AuNPs treatment in IPA mice. Effect LH-AuNPs on the antioxidant enzymes production, including (**D**) CAT, (**E**) SOD, and (**F**) MDA in the lung of IPA mice after 3 days of LH-AuNPs treatment. Data are expressed as means ± SD (n = 10 mice/group). (* *p* < 0.05 and ** *p* < 0.005, compared to control non-treated mice).

As shown in Figure 7A–C, the elevated levels of pro-inflammatory cytokines, including TNFα, IL-1, and IL-17, in IPA mice were significantly reduced in LH-AuNPs-treated mice (Figure 7A–C). Measurement of antioxidant enzymes activities in the lung of non-treated IPA mice revealed the reduced levels of both CAT and SOD enzymes while increased levels of MDA enzyme (Figure 7A–C). In contrast, IPA mice treated with LH-AuNPs displayed increased levels of both CAT and SOD and reduced levels of MDA as compared to IPA-non-treated mice group (Figure 7D–F).

## 4. Discussion

In this study, we used for the first time the leaf extract of *L. hastate* to biosynthesize AuNPs with high antifungal activity against *A. fumigatus* in vitro and in vivo.

We biosynthesized LH-AuNPs using ethanol extract of *L. hastate* as a green reductant, which improves biocompatibility and pharmacological potential. In consistency with our procedure, it was reported that once the AuNPs are formed in a reaction mixture, the color changes to a ruby red occur because of the surface plasmon resonance (SPR) [36,37]. The absorption peak of the UV spectrum for the biosynthesized LH-AuNPs was 544 nm, which is almost the same absorbance band (at 549 nm) of synthesized AuNPs using extract of *Crocus sativus* [38]. 

TEM displayed LH-AuNPs particles about 5–30 nm in size with spherical-, hexagonal-, and triangular-shaped appearances. AuNPs with these distinctive structures have previously been described [39]. In addition, the EDX and XRD analyses verified the presence of AuNPs and their typical metallic gold nano-crystalline structural configurations. It was reported that both the hydroxyl and carbonyl groups are accountable for the stabilization of gold nanoparticles [40]. Our FTIR spectra displayed peaks related to OH and carbonyl groups capped the nanoparticle surfaces. These results are in agreement with reported results of biosynthesized AuNPs using *Tetraselmis suesica* [41]. 

In this study, we examined the antifungal activity of LH-AuNPs against *A. fumigatus* using an MTT assay. Our results showed that the MIC of LH-AuNPs was 64 μg/mL. Green synthesized AuNPs using different plant extracts showed remarkable antifungal activity against *Aspergillus* species. For example, the results of the serial dilution plate counting method revealed that the biosynthesized AuNPs using *Viola betonicifolia* extract significantly inhibited the growth of *A. fumigatus*, *A. flavus*, and *A. niger* with efficacy greater than 67% [42]. The results of the agar well diffusion method showed that biosynthesized AuNPs using *Mentha piperita* extract were highly active against *A. flavus* with an inhibition zone diameter (IZD) of 18.49 mm [43]. Similarly, the antifungal potential of licorice-root-extract-mediated synthesis of AuNPs was very high towards *A. flavus* with IZD of 18 mm [44].

Our results showed that the LH-AuNPs have moderate antifungal action against *A. fumigatus* when compared to AMB. Although AMB exhibited high antifungal activity via interacting with sterols of mammalian cells, AMB caused several side effects in treated patients, including higher rates of nephrotoxicity and hypokalemia [45]. Furthermore, only 50% of patients with invasive fungal aspergillosis showed a promising response to AMB treatment, and the survival rates were approximately 59%. On the other hand, AuNPs are recommended for treatment due to their basic antifungal potential or as a drug delivery vehicle with an emphasis on decreasing the dose of antifungal agent required for treatment [46].

Interestingly, the leaf extract of *L. hastate* displayed significant antifungal potential against different fungal species, including *A. flavus* and *A. niger*, due to the presence of biologically active antifungal compounds [47,48]. These include sterols, terpenoids, alkaloids, flavonoids, phenols, carbohydrates, tannins, Proanthocyanidins, and glycosides [49,50]. 

The mechanism of the antifungal action of gold ions implicates their absorption and accumulation by the fungal cell, which in turn damages the cell membrane via inhibiting the cell wall β-glucan synthase. Moreover, AuNPs might interact with cell wall macromolecules and membrane proteins [51]. In this context, our SEM and TEM images showed significant morphological alterations in *A. fumigatus* upon exposure to AuNPs. The ultrastructural modifications include disturbing cell permeability by making pits and gaps in the cell membrane, which leads to structural changes in the outer membrane and fungal cell death. These data are consistent with the reported effects of AuNPs on *C. albicans* to cause collapse and disintegration of the cytoplasm material [52,53].

In this study, *A. fumigatus* treated with LH-AuNPs displayed a loss of green pigmentation. Melanin pigments improve the ability of fungi to resist immune clearance, increase virulence and drug resistance, and favor pathogenicity [54]. In *A. fumigatus*, piomelanins protect hyphal cell walls from ROS and gray-green. DHN-melanins maintain the structural integrity of the cell wall of conidia due to their adhesive properties [55]. In this context, the reduced virulence toward mice was reported for non-melanizing (white) mutants of *A. fumigatus* [56], while non-pigmented mutant of *A. fumigatus* was more susceptible to being eliminated by human monocytes as compared to wild-type conidia [57]. 

Our data were the first to demonstrate the low cytotoxicity of LH-AuNPs on cultured primary BMSCs and MCS5. AuNPs showed different toxicity effects on different cell types; for example, AuNPs induced oxidative-stress-related cytotoxicity in hepatocytes [58] and small airway epithelial cells (SAECs) [59]. On the other hand, Au-NPs caused low long toxicity effect on several other cell lines, including MG63 cells, HeLa cells, and Vero cells [60,61].

We demonstrated the effective treatment of IPA mice with intravenous injection of LH-AuNPs without observing any in vivo cytotoxicity. Many reports investigated the in vivo pharmacokinetic profile of AuNPs after intravenous administration and showed the clearance of Au-NPs from the bloodstream and its accumulation preferentially in the liver, kidney, and spleen. However, these accumulations of AuNPs did not cause any hepatic or renal toxicity [62,63,64]. In consistent, our results demonstrated that LH-AuNPs do not cause any acute or chronic toxicity in vivo. 

Production of gliotoxin, an immune-suppressive mycotoxin, is involved in the pathogenesis of IPA and might be a marker of infection with *A. fumigatus* [65,66]. In this context, our data demonstrated the direct effect of LH-AuNPs administration on inhibiting the *A. fumigatus* burden by more than 60% and suppressing their gliotoxin production. 

The mouse model of IPA demonstrated activation of inflammatory programs by increasing inflammatory cytokine-mediated pathology, including production of IL-1, IL-17, IL-23, and TNF by eosinophils, inflammatory monocytes, dendritic cells (DCs), and alveolar macrophages [67,68,69,70]. Our results demonstrated the effective inhibitory effect of LH-AuNPs on IPA-induced inflammatory cytokines. Similarly, we have recently reported the therapeutic potential of a single dose of green synthesized AS-AgNPs to suppress IPA-induced pro-inflammatory cytokines and lung tissue damage in mice [14]. The anti-inflammatory properties of Au-NPs were reported in vitro and in vivo in many studies to be mediated by antioxidant properties [71,72]. AuNPs can act to reduce the production of reactive oxygen and display good free radical scavenging activity against antioxidant enzymes [73]. In addition, the anti-inflammatory properties of Au compounds, which are used for the treatment of inflammatory disorders (such as rheumatoid arthritis, stroke, and cerebral damage), were found to be mediated by inhibiting the activation of nuclear factor kappa-light-chain-enhancer of activated B cells (NF-κB) [74,75,76].

## 5. Conclusions

The green synthesized LH-AuNPs showed significant antifungal activity against *A. fumigatus*, including the inhibition of fungal hyphae radial growth and green pigmentation and the deterioration of cell walls. Intravenous administration of LH-AuNPs in the IPA mice model displayed obvious therapeutic potential for lung repair without showing any in vivo cytotoxicity. The therapeutic potential of LH-AuNPs in vivo was found to be mediated via the inhibitory effect of LH-AuNPs on the fungal burden, gliotoxin production, and IPA-induced inflammatory cytokines production in the lung of IPA mice.

## Data Availability

All materials are available by the corresponding author.

## Supplementary Materials

The following supporting information can be downloaded at: https://www.mdpi.com/article/10.3390/jof8050442/s1, Figure S1: FTIR spectrum of *L. hastata* leaf extract; Figure S2: Zeta potential of LH-AuNPs.

## References

[1] Zarrinfar H., Mirhendi H., Fata A., Khodadadi H., Kordbacheh P. (2015). Detection of Aspergillus flavus and A. fumigatus in Bronchoalveolar Lavage Specimens of Hematopoietic Stem Cell Transplants and Hematological Malignancies Patients by Real-Time Polymerase Chain Reaction, Nested PCR and Mycological Assays. Jundishapur J. Microbiol..

[2] Kashefi E., Seyedi S.J., Zomorodian K., Zare Shahrabadi Z., Zarrinfar H. (2021). Successful treatment of pulmonary aspergillosis due to Aspergillus fumigatus in a child affected by systemic lupus erythematosus: A case report from Northeastern Iran. Clin. Case Rep..

[3] Seidler M.J., Salvenmoser S., Müller F.M. (2008). Aspergillus fumigatus forms biofilms with reduced antifungal drug susceptibility on bronchial epithelial cells. Antimicrob. Agents Chemother..

[4] Chen J., Yang Q., Huang J., Li L. (2013). Risk factors for invasive pulmonary aspergillosis and hospital mortality in acute-on-chronic liver failure patients: A retrospective-cohort study. Int. J. Med. Sci..

[5] Groll A.H., Piscitelli S.C., Walsh T.J. (1998). Clinical pharmacology of systemic antifungal agents: A comprehensive review of agents in clinical use, current investigational compounds, and putative targets for antifungal drug development. Adv. Pharmacol..

[6] Jenks J.D., Hoenigl M. (2018). Treatment of Aspergillosis. J. Fungi.

[7] Correa-Royero J., Tangarife Castaño V., Durán D., Stashenko E., Mesa A. (2010). In vitro antifungal activity and cytotoxic effect of essential oils and extracts of medicinal and aromatic plants against Candida krusei and Aspergillus fumigatus. Rev. Bras. De Farmacogn..

[8] Connor E.E., Mwamuka J., Gole A., Murphy C.J., Wyatt M.D. (2005). Gold nanoparticles are taken up by human cells but do not cause acute cytotoxicity. Small.

[9] Dreaden E.C., Alkilany A.M., Huang X., Murphy C.J., El-Sayed M.A. (2012). The golden age: Gold nanoparticles for biomedicine. Chem. Soc. Rev..

[10] Pissuwan D., Niidome T., Cortie M.B. (2011). The forthcoming applications of gold nanoparticles in drug and gene delivery systems. J. Control. Release Off. J. Control. Release Soc..

[11] De Jong W.H., Borm P.J.A. (2008). Drug delivery and nanoparticles:applications and hazards. Int. J. Nanomed..

[12] Sibuyi N.R.S., Moabelo K.L., Fadaka A.O., Meyer S., Onani M.O., Madiehe A.M., Meyer M. (2021). Multifunctional Gold Nanoparticles for Improved Diagnostic and Therapeutic Applications: A Review. Nanoscale Res. Lett..

[13] Zhang D., Ma X.-L., Gu Y., Huang H., Zhang G.-W. (2020). Green Synthesis of Metallic Nanoparticles and Their Potential Applications to Treat Cancer. Front. Chem..

[14] Ali E.M., Abdallah B.M. (2021). Effective Inhibition of Invasive Pulmonary Aspergillosis by Silver Nanoparticles Biosynthesized with Artemisia sieberi Leaf Extract. Nanomaterials.

[15] Abdallah B.M., Ali E.M. (2021). Green Synthesis of Silver Nanoparticles Using the Lotus lalambensis Aqueous Leaf Extract and Their Anti-Candidal Activity against Oral Candidiasis. ACS Omega.

[16] Freiberger C.E., Vanderjagt D.J., Pastuszyn A., Glew R.S., Mounkaila G., Millson M., Glew R.H. (1998). Nutrient content of the edible leaves of seven wild plants from Niger. Plant Foods Hum. Nutr..

[17] Sena L.P., Vanderjagt D.J., Rivera C., Tsin A.T., Muhamadu I., Mahamadou O., Millson M., Pastuszyn A., Glew R.H. (1998). Analysis of nutritional components of eight famine foods of the Republic of Niger. Plant Foods Hum. Nutr..

[18] Mansurah A., Salisu Maiwada A., Sa’id I., Jafar Musa M.A. (2017). Isolation and Characterization of a Potential Angiotensin-Converting Enzyme Inhibitory Peptide from the Leaves of Leptadenia hastata (Asclepiadaceae). Malays. J. Appl. Sci..

[19] Malgwi S.A., Zango M.K., Mbaya A.W., Dennis G., Kyari F., Sanda K.A., Balami S.B., Bwala A.D. (2019). Anti-trypanosomal activity of crude root extract of Leptadenia hastata (Pers) dec.ne in Wistar rats infected with Trypanosoma brucei brucei and associated hematological changes. J. Adv. Vet. Anim. Res..

[20] Umaru I. (2017). Antifungal Activity of Leptadenia hastata (Pers) Decne Leaves Extract. Int. J. Pure Appl. Biosci..

[21] Umaru I. (2018). Phytochemical, antifungal and antibacterial potential of Leptadenia hastata stem-bark extract. MOJ Toxicol..

[22] Manavathu E.K., Cutright J.L., Loebenberg D., Chandrasekar P.H. (2000). A comparative study of the in vitro susceptibilities of clinical and laboratory-selected resistant isolates of Aspergillus spp. to amphotericin B, itraconazole, voriconazole and posaconazole (SCH 56592). J. Antimicrob. Chemother..

[23] Meletiadis J., Meis J.F., Mouton J.W., Donnelly J.P., Verweij P.E. (2000). Comparison of NCCLS and 3-(4,5-dimethyl-2-Thiazyl)-2, 5-diphenyl-2H-tetrazolium bromide (MTT) methods of in vitro susceptibility testing of filamentous fungi and development of a new simplified method. J. Clin. Microbiol..

[24] Kaur P., Thakur R., Chaudhury A. (2012). An in vitro study of the antifungal activity of silver/chitosan nanoformulations against important seed borne pathogens. Int. J. Sci. Technol. Res..

[25] Achar P.N., Quyen P., Adukwu E.C., Sharma A., Msimanga H.Z., Nagaraja H., Sreenivasa M.Y. (2020). Investigation of the Antifungal and Anti-Aflatoxigenic Potential of Plant-Based Essential Oils against Aspergillus flavus in Peanuts. J. Fungi.

[26] Sbaraglia G., D’Errico P., Serafini S., Vecchiarelli L., Perito S. (1984). Pathogenicity of various species of Candida in mice immunodepressed with cyclophosphamide. Boll. Della Soc. Ital. Di Biol. Sper..

[27] Fahmy S.R., Ali E.M., Ahmed N.S. (2014). Therapeutic effect of Sepia ink extract against invasive pulmonary aspergillosis in mice. J. Basic Appl. Zool..

[28] Botelho D., Leo B.F., Massa C., Sarkar S., Tetley T., Chung K.F., Chen S., Ryan M.P., Porter A., Atochina-Vasserman E.N. (2018). Exposure to Silver Nanospheres Leads to Altered Respiratory Mechanics and Delayed Immune Response in an in Vivo Murine Model. Front. Pharmacol..

[29] Boudra H., Morgavi D. (2005). Mycotoxin risk evaluation in feeds contamined by Aspergillus fumigatus. Anim. Feed Sci. Technol..

[30] Abdallah B.M., Alzahrani A.M., Abdel-Moneim A.M., Ditzel N., Kassem M. (2019). A simple and reliable protocol for long-term culture of murine bone marrow stromal (mesenchymal) stem cells that retained their in vitro and in vivo stemness in long-term culture. Biol. Proced. Online.

[31] Abdallah B.M. (2019). Ali EM: 5′-hydroxy Auraptene stimulates osteoblast differentiation of bone marrow-derived mesenchymal stem cells via a BMP-dependent mechanism. J. Biomed. Sci..

[32] Teranishi Y., Tanaka A., Osumi M., Fukui S. (1974). Catalase Activities of Hydrocarbon-utilizing Candida Yeasts. Agric. Biol. Chem..

[33] McCord J.M., Fridovich I. (1969). Superoxide dismutase. An enzymic function for erythrocuprein (hemocuprein). J. Biol. Chem..

[34] Ohkawa H., Ohishi N., Yagi K. (1979). Assay for lipid peroxides in animal tissues by thiobarbituric acid reaction. Anal. Biochem..

[35] Das T., Mishra S., Nag S., Saha K.D. (2022). Green-synthesized gold nanoparticles from black tea extract enhance the chemosensitivity of doxorubicin in HCT116 cells via a ROS-dependent pathway. RSC Adv..

[36] Hu X., Zhang Y., Ding T., Liu J., Zhao H. (2020). Multifunctional Gold Nanoparticles: A Novel Nanomaterial for Various Medical Applications and Biological Activities. Front. Bioeng. Biotechnol..

[37] Ahn S., Singh P., Jang M., Kim Y.J., Castro-Aceituno V., Simu S.Y., Kim Y.J., Yang D.C. (2018). Gold nanoflowers synthesized using Acanthopanacis cortex extract inhibit inflammatory mediators in LPS-induced RAW264.7 macrophages via NF-κB and AP-1 pathways. Colloids Surf. B Biointerfaces.

[38] Das R.K., Babu P.J., Gogoi N., Sharma P., Bora U. (2012). Microwave-Mediated Rapid Synthesis of Gold Nanoparticles Using *Calotropis procera* Latex and Study of Optical Properties. ISRN Nanomater..

[39] Salunke G.R., Ghosh S., Santosh Kumar R.J., Khade S., Vashisth P., Kale T., Chopade S., Pruthi V., Kundu G., Bellare J.R. (2014). Rapid efficient synthesis and characterization of silver, gold, and bimetallic nanoparticles from the medicinal plant Plumbago zeylanica and their application in biofilm control. Int. J. Nanomed..

[40] Shervani Z., Taisuke Y., Ifuku S., Saimoto H., Morimoto M. (2012). Preparation of Gold Nanoparticles Loaded Chitin Nanofiber Composite. Adv. Nanopart..

[41] Shakibaie M., Forootanfar H., Mollazadeh-Moghaddam K., Bagherzadeh Z., Nafissi-Varcheh N., Shahverdi A.R., Faramarzi M.A. (2010). Green synthesis of gold nanoparticles by the marine microalga Tetraselmis suecica. Biotechnol. Appl. Biochem..

[42] Wang M., Meng Y., Zhu H., Hu Y., Xu C.P., Chao X., Li W., Li C., Pan C. (2021). Green Synthesized Gold Nanoparticles Using Viola betonicifolia Leaves Extract: Characterization, Antimicrobial, Antioxidant, and Cytobiocompatible Activities. Int. J. Nanomed..

[43] Thanighaiarassu R.R., Sivamai P., Devika R., Nambikkairaj B. (2014). Green Synthesis of Gold Nanoparticles Characterization by using Plant Essential Oil Menthapiperita and their Antifungal Activity against Human Pathogenic Fungi. J. Nanomed. Nanotechnol..

[44] Al-Radadi N.S. (2021). Facile one-step green synthesis of gold nanoparticles (AuNp) using licorice root extract: Antimicrobial and anticancer study against HepG2 cell line. Arab. J. Chem..

[45] Carmona E.M., Limper A.H. (2017). Overview of Treatment Approaches for Fungal Infections. Clin. Chest Med..

[46] Zhao L., Seth A., Wibowo N., Zhao C.X., Mitter N., Yu C., Middelberg A.P. (2014). Nanoparticle vaccines. Vaccine.

[47] Umaru I., Ahmad F., Wakawa H., Aduwamai U., Umaru K. (2018). Antifungal Potential of Leptadenia Hastata Against Some Pathogenic Fungi. Am. J. Biochem. Biotechnol..

[48] Bayala B., Maria H., Ouédraogo A., Keiler A., Tamboura H. (2018). Leptadenia hastata Pers. (Decne) a Promising Source for Natural Compounds in Biomedical Applications. Am. J. Drug Discov. Dev..

[49] Imam I.U., Salihu Abdallah M., Ali M. (2019). Antibacterial Activity of Leptadenia Hastata Leaves Extracts against Some Gastro-Intestinal Isolates. Arch. Biomed. Eng. Biotechnol..

[50] Haruna A., Mann A., Ogbadoyi E.O. (2018). Phytochemical composition and antitrypanosomal activity of the leaf extract of Leptadenia hastata (Pers) Decne. Bayero J. Pure Appl. Sci..

[51] Gutiérrez J.A., Caballero S., Díaz L.A., Guerrero M.A., Ruiz J., Ortiz C.C. (2018). High Antifungal Activity against Candida Species of Monometallic and Bimetallic Nanoparticles Synthesized in Nanoreactors. ACS Biomater. Sci. Eng..

[52] Hamad K., Mahmoud N., Al-Dabash S., Abd Al-Samad L., Abdallah M., Al-Bakri A. (2020). Fluconazole conjugated-gold nanorods as an antifungal nanomedicine with low cytotoxicity against human dermal fibroblasts. RSC Adv..

[53] Tharwat N., Al-Bedak O., Hamouda R., Shreif R., Mounir R., Sami A. (2019). Antifungal effect of gold nanoparticles on fungi isolated from onychomycosis patients. Al-Azhar J. Pharm. Sci..

[54] Belozerskaya T., Gessler N., Aver‘yanov A. (2015). Melanin Pigments of Fungi.

[55] Pihet M., Vandeputte P., Tronchin G., Renier G., Saulnier P., Georgeault S., Mallet R., Chabasse D., Symoens F., Bouchara J.-P. (2009). Melanin is an essential component for the integrity of the cell wall of Aspergillus fumigatus conidia. BMC Microbiol..

[56] Jacobson E.S. (2000). Pathogenic roles for fungal melanins. Clin. Microbiol. Rev..

[57] Liu G.Y., Nizet V. (2009). Color me bad: Microbial pigments as virulence factors. Trends Microbiol..

[58] Li J.J., Hartono D., Ong C.N., Bay B.H., Yung L.Y. (2010). Autophagy and oxidative stress associated with gold nanoparticles. Biomaterials.

[59] Ng C.-T., Li J., Gurung R., Hande P., Ong C.-N., Bay B.-H., Yung L.-Y. (2013). Toxicological profile of small airway epithelial cells exposed to gold nanoparticles. Exp. Biol. Med..

[60] Sani A., Cao C., Cui D. (2021). Toxicity of gold nanoparticles (AuNPs): A review. Biochem. Biophys. Rep..

[61] Patra H.K., Banerjee S., Chaudhuri U., Lahiri P., Dasgupta A.K. (2007). Cell selective response to gold nanoparticles. Nanomed. Nanotechnol. Biol. Med..

[62] Bailly A.L., Correard F., Popov A., Tselikov G., Chaspoul F., Appay R., Al-Kattan A., Kabashin A.V., Braguer D., Esteve M.A. (2019). In vivo evaluation of safety, biodistribution and pharmacokinetics of laser-synthesized gold nanoparticles. Sci. Rep..

[63] Zhang J., Nie X., Ji Y., Liu Y., Wu X., Chen C., Fang X. (2014). Quantitative Biokinetics and Systemic Translocation of Various Gold Nanostructures Are Highly Dependent on Their Size and Shape. J. Nanosci. Nanotechnol..

[64] Wang J., Bai R., Yang R., Liu J., Tang J., Liu Y., Li J., Chai Z., Chen C. (2015). Size- and surface chemistry-dependent pharmacokinetics and tumor accumulation of engineered gold nanoparticles after intravenous administration. Met. Integr. Biometal Sci..

[65] Hof H., Kupfahl C. (2009). Gliotoxin in Aspergillus fumigatus: An example that mycotoxins are potential virulence factors. Mycotoxin Res..

[66] Gayathri L., Akbarsha M.A., Ruckmani K. (2020). In vitro study on aspects of molecular mechanisms underlying invasive aspergillosis caused by gliotoxin and fumagillin, alone and in combination. Sci. Rep..

[67] Amarsaikhan N., Tsoggerel A., Hug C., Templeton S.P. (2019). The Metabolic Cytokine Adiponectin Inhibits Inflammatory Lung Pathology in Invasive Aspergillosis. J. Immunol..

[68] Schelenz S., Smith D.A., Bancroft G.J. (1999). Cytokine and chemokine responses following pulmonary challenge with Aspergillus fumigatus: Obligatory role of TNF-alpha and GM-CSF in neutrophil recruitment. Med. Mycol..

[69] Heinekamp T., Schmidt H., Lapp K., Pähtz V., Shopova I., Köster-Eiserfunke N., Krüger T., Kniemeyer O., Brakhage A.A. (2015). Interference of Aspergillus fumigatus with the immune response. Semin. Immunopathol..

[70] Al-Bader N., Sheppard D.C. (2016). Aspergillosis and stem cell transplantation: An overview of experimental pathogenesis studies. Virulence.

[71] Zhu S., Jiang X., Boudreau M.D., Feng G., Miao Y., Dong S., Wu H., Zeng M., Yin J.-J. (2018). Orally administered gold nanoparticles protect against colitis by attenuating Toll-like receptor 4- and reactive oxygen/nitrogen species-mediated inflammatory responses but could induce gut dysbiosis in mice. J. Nanobiotechnol..

[72] Haupenthal D., Possato J.C., Zaccaron R.P., Mendes C., Rodrigues M.S., Nesi R.T., Pinho R.A., Feuser P.E., Machado-de-Ávila R.A., Comim C.M. (2020). Effects of chronic treatment with gold nanoparticles on inflammatory responses and oxidative stress in Mdx mice. J. Drug Target.

[73] Narayanan K.B., Park H.H. (2013). Pleiotropic functions of antioxidant nanoparticles for longevity and medicine. Adv. Colloid Interface Sci..

[74] Jeon K.-I., Byun M.-S., Jue D.-M. (2003). Gold compound auranofin inhibits IκB kinase (IKK) by modifying Cys-179 of IKKβ subunit. Exp. Mol. Med..

[75] Fujita T., Zysman M., Elgrabli D., Murayama T., Haruta M., Lanone S., Ishida T., Boczkowski J. (2021). Anti-inflammatory effect of gold nanoparticles supported on metal oxides. Sci. Rep..

[76] Khan M.A., Khan M.J. (2018). Nano-gold displayed anti-inflammatory property via NF-kB pathways by suppressing COX-2 activity. Artif. Cells Nanomed. Biotechnol..

