# Supplementary material for: Therapeutic Potential of Green Synthesized Gold Nanoparticles Using Extract of Leptadenia hastata against Invasive Pulmonary Aspergillosis
Source: J Fungi (Basel). 2022 Apr 24;8(5):442. doi: 10.3390/jof8050442 (PMC9146234; doi:10.3390/jof8050442)
Supplement: Supplementary file 1 [file jof-08-00442-s001.zip › jof-1676257-supplementary.pdf]

# Supplementary figure S1

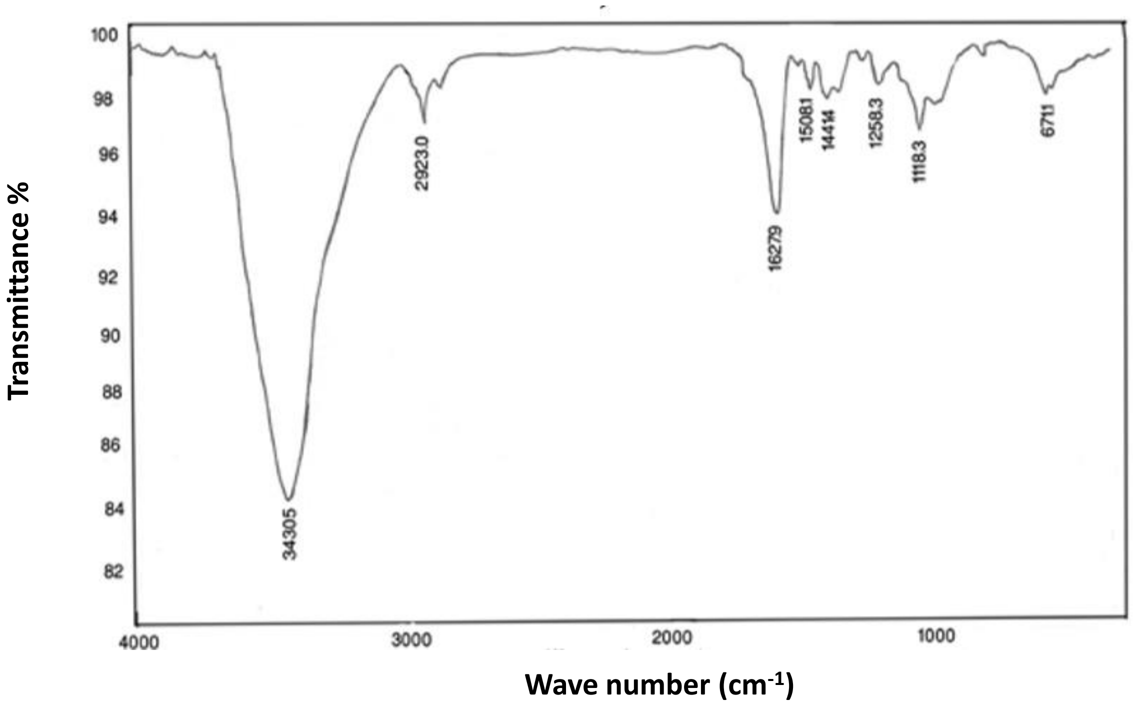


**Supplementary Figure S1.** FTIR spectrum of *L. hastata* leaf extract exhibited two peaks related to OH/ NH and C=O groups. The presence of OH group could be related to peak at 3430.5  cm-1. The peak of 1627.9 was corresponded to C=O group.

# Supplementary figure S2

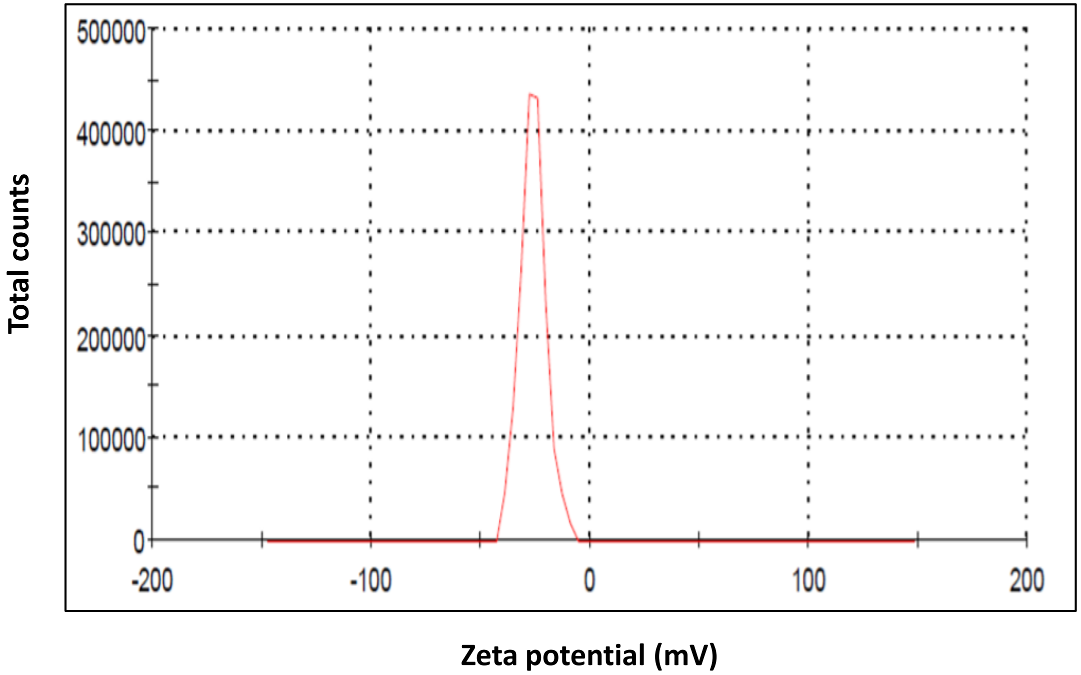


**Supplementary Figure S2.** Zeta potential of LH-AuNPs
